# Determining optimal laser-beam cutting equipment investment through a robust optimization modeling approach

**DOI:** 10.1371/journal.pone.0254893

**Published:** 2021-07-23

**Authors:** Juan Feller, Alejandro F. Mac Cawley, Jorge A. Ramos-Grez, Iván La Fé-Perdomo

**Affiliations:** 1 Department of Industrial and Systems Engineering, Pontificia Universidad Católica de Chile, Santiago, Chile; 2 Department of Mechanical and Metallurgical Engineering, Pontificia Universidad Católica de Chile, Santiago, Chile; 3 Centre for Advanced and Sustainable Manufacturing Studies, University of Matanzas, Matanzas, Cuba; J.C. Bose University of Science and Technology, YMCA, INDIA, INDIA

## Abstract

The acquisition of Advanced Manufacturing Technologies (AMT), such as high-power fiber or *CO*_2_ laser cutting equipment, generally involves high investment levels. Its payback period is usually more extended, and there is a moderate-to-high risk involved in adopting these technologies. In this work, we present a robust model that optimizes equipment investing decisions, considers the process’s technical constraint and finds an optimal production plan based on the available machinery. We propose a linear investment model based on historical demand information and take physical process parameters for a LASER cutting equipment, such as cutting speed and gas consumption. The model is then transformed into a robust optimization model which considers demand uncertainty. Second, we determine the optimal production plan based on the results of the robust optimization model and assuming that demand follows a normal distribution. As a case study, we decided on the investment and productive plan for a company that offers Laser-Beam Cutting (LBC) services. The case study validates the effectiveness of the proposed model and proves the robustness of the solution. For this specific application of the model, results showed that the optimal robust solution could increase the company’s expected profits by 6.4%.

## 1 Introduction

Investment in new technologies, such as AMT, has become a requirement for companies to stay competitive. Benefits from investing in AMT arise from: reduced inventory, less floor space, improved return on equity, reduction in unit production cost and cycle time, increased flexibility, improved product quality, increased productivity, quick response to customer demand, increased ease of operation, and enhanced employee relationship [1–6]. LASER beam cutting, in particular of metals, has been on the market for over 40 years. Some of its advantages consider high cutting speeds and dimensional precision as well as complex geometry contouring, ease of automation through the use of computer numerical control (CNC), and the fact that the LASER is considered a green manufacturing tool [7–9]. Among its limitations can be found difficulty in cutting sheets of large thicknesses (e.g., above 25 mm), processing reflective materials such as copper and its alloys, and the high investment cost associated with it [10].

To remain competitive, companies must improve their productivity, reducing fixed and variable costs, which the investment in new manufacturing technologies can achieve [11, 12]. However, decision-makers need to ponder several complex factors at the moment of deciding the adequate machinery and the optimal moment to invest in [13]. Many aspects need to be considered, among which one finds: product demand, quality, cost, production efficiency, and capital expenditure. An important issue, like setup flexibility, is sometimes neglected during the acquisition process. This factor determines the ability of the equipment to perform cost-effectively and rapidly a set of different tasks [1, 14–17]. Gorissen et al. [18] indicates that there is a significant potential for real-life applications of robust optimization, and Slack [19] states that flexibility is an important concept that must be taken into account in productive industries due to the lack of stability and predictability of the environment. Thus, manufacturing equipment must be flexible enough to allow mass production efficiencies [16, 20, 21]. Hence, making the right equipment type acquisition decision at the right time will provide considerable operational and competitive benefits for the enterprise [22, 23].

In the AMT literature, optimization has been mainly focused on modeling the process parameters and their interactions, which generally involves selecting the exact parameters settings that considerably affect its performance. Examples of process parameter optimization can be found in electrical discharge machining (EDM) [24, 25], grinding processes [26, 27], and cutting processes [28]. See Rao & Kalyankar for an in-depth review of process optimization [29]. In the equipment selection methods, most of the research in AMT has been focused on multi-criteria decision-making methods [30–32] or fuzzy techniques [33–36]. Optimization approaches to equipment selection have been previously used in the mining context [37, 38] and in the print production environments [39]. However, none of them has taken into account demand uncertainty in their decision-making process. Regarding the optimization of the production decision parameters, the investigations have been focused on technical aspects of the process parameters [40].

Optimization models are frequently used across all companies in the manufacturing industry. Although models are exposed to different sources of uncertainty, some companies decide to optimize production assuming that all information is known; this is not adequate for real problems [41, 42]. The literature shows that there are two main approaches to incorporate data uncertainty in a single and multi-period decision-making process; these are stochastic programming (SP) and robust optimization (RO) [18, 43–45].

Stochastic programming (SP) is a rigorous approach that assumes that the probability distributions of the uncertain parameters are known and can be accurately estimated. This approach requires an accurate probabilistic description of the variables and data, which in some cases is very difficult to obtain [45, 46]. Stochastic programming is the most complex and challenging method for managers to understand [43]. Its use has been widely reported in the literature [47, 48], in fields such as system capacity expansion, portfolio selection, scheduling maintenance personnel, investment decision, among others.

On the other hand, robust optimization (RO) is a relatively new methodology that includes uncertainty in the problem model. Its goal is to obtain solutions that remain feasible for any uncertain outcome of a given set of parameters [42, 44, 49–52]. This approach accounts for variations in the optimal results caused by fluctuations on the parameters [53]. Thus, it delivers solutions that are less sensitive to the variations of the parameters [54]. The RO methodology generates solutions that are progressively less sensitive to data uncertainty; it does not need detailed probabilistic knowledge, nor specific distributions of the uncertain parameters [18, 55]. Unlike stochastic programming, RO has been more extensively used as it requires less information and still accounts for uncertainty. Nonetheless, it is essential to mention that RO is complex to implement and requires more computational power than other methodologies [55].

The RO methodology was first developed by [56]. The latter proposes a deterministic linear optimization model which finds solutions that will always be feasible for all the data points in a convex set. So, in a RO approach, the decision-maker needs to determine the level of robustness or class in which the problem will continue to be feasible under an uncertain outcome of a given set of parameters [42, 43, 49]. The RO approach requires additional inputs called uncertainty budgets, which should be based on the decision-makers’ risk aversion since these inputs determine the conservatism of the solution. The impact of uncertainty budget variations on the problem-solution is not obvious, and the results’ analysis tends to be more complicated. Several authors describe the structure that uncertainty sets must-have for the RO outputs to be accurate and reliable. In addition, the literature defines different types of reformulations for constraints that include uncertain variables or parameters. [41] proposes a robust optimization with a polyhedral uncertainty set for a Sawmill planning problem where the uncertainty lies in the yield parameter for the cutting process. The same approach is used by [43] who presents a robust optimization model for a Sawmill production scheduling under uncertainty in product demand and raw materials supply. In both examples, the authors evaluate the robustness and conservatism level of the solution, providing several managerial insights that could help production schedulers to choose the appropriate level of conservatism based on the uncertainty they are facing. [57] compare three uncertainty sets (i.e., Box, Ellipsoidal, and Convex Hull) for a vehicle routing problem with uncertain demand. The conclusions are that box uncertainty sets lead to the most unfilled demand and highest cost; convex hull uncertainty sets result in the lowest value of the objective function, and ellipsoidal uncertainty sets generate an objective function value greater than the other two approaches. In [57]’s model, all uncertainty behave with similar trends, and the chosen uncertainty set causes the differences.

In the case of a specific AMT technology, such as LASER-Beam Cutting (LBC), the optimization literature has mostly focused on optimizing the machine’s parameters to maximize cutting process effectiveness, and efficiency [29]. For more details on the LASER cutting process, we recommend reviewing [7, 8, 10, 58, 59]. However, it is important to consider that the main differences between LASER technologies today are: type of active medium, beam power, and the materials that can be processed. *CO*_2_ LASER is an electrically pumped gas LASER that radiates light at a wavelength of 10.6 *μ*m [60]. It is used for fine cutting of metal sheets at high speeds due to its high average beam power, improved efficiency, and beam mode quality, which allows focusing the energy down to small spot size. Nd:YAG LASER is an optically pumped solid state LASER that operates at a wavelength of 1.06 *μ*m [61]. Due to its shorter wavelength, this LASER is more suitable for processing reflective metals such as aluminum, copper, and steel. On the other hand, the fiber LASER is especially suited for efficient material treatment due to its high beam quality and high power efficiency of up to 40%. In this type of LASER, the radiation of the light is guided by a light-conducting cable [60]. [61] develop a model to optimize the main parameters for LASER cutting machines, as cutting speed, beam power, and gas pressure.

Our contribution is to propose and solve a linear robust optimization model which accounts for the demand variability and uses physical process parameters for LASER cutting equipment such as cutting speed and gas consumption. The latter to determine the optimal equipment selection and production planning. In this work, we use a robust optimization approach that takes into account the parameters ambiguity and stochastic uncertainty [62]. The model produces an optimal investment plan for a defined period and the adequate equipment selection required to prevent gaps in supply due to demand fluctuations. We compare the results generated by a linear optimization model with a robust optimization to understand the effects of demand uncertainty in the equipment selection and production planning process. To validate our findings, we present the case study of a company that offers LASER-Beam Cutting (LBC) services.

The rest of this paper is structured as follows: in section two, the model, variables involved, and the mathematical formulation for the optimization problem is explained. In section three, we describe the robust optimization methodology based on the approach of Bertsimas and Melvyn [49]. Section four presents a case study where we apply the proposed model to a LASER cutting services company. In section five, we analyze the model results, discussing the level of robustness and its implications. Finally, we outline some conclusions and present potential further research that could be continued on this topic.

## 2 Equipment investment and production optimization planning model

To determine the optimal equipment selection and production planning for an AMT LASER cutting company, we have divided the problem into two integrated decision models: first, an investment and second, a production planning model. The investment model tries to determine the best selection of LBC technology and the moment to make the investment decision to increase the production capacity of a company that manufactures (*p*) different types of cut-based products. Based on the machine configuration generated by the first model, the second model determines the best production strategy that maximizes the company’s economic benefits.

There are (*m*) different types of machinery for our case study, each with different technical features. Each machine was modelled using two parameters that directly affect the production process. The first parameter is the production rate (cutting velocity) ($\nu_{p}^{m}$), which represent how many products can be cut in a certain period of time. This parameter varies depending on the products and the cutting machine. The second one is the production cost ($G_{p}^{m}$) per time unit, which represent the amount of money that the company has to pay for a machine to cut a specific product on a period. (*α*_*p*_) represents the price of each minute of production for a specific item.

On each lapsus of time, the company faces a specific demand (*D*_*p*,*t*_) for each product. The request must be filled to avoid a monetary penalization of (*ρ*_*p*_) for not filling it (penalization is different for each product). The company can invest only in one cutting machine per time, which will be operational at the beginning of the next period. Each machine has a value of (*γ*^*m*^) and can only cut a certain set of products.

We will now describe the technical parameters that govern the laser beam cutting process and then develop the investment and production model in relation to this advanced manufacturing process.

### 2.1 Laser cutting machine parameters: Cutting speed and gas consumption

The performance of a LBC machine mainly depends on laser parameters (e.g. laser power, wavelength, mode of operation: pulsed or continuous), material parameters (e.g. type, thickness), and process parameters (e.g. cutting speed, focal plane position, frequency, energy, pulse duration, assist gas type and pressure) [63]. We will focus on 2-D cutting laser machines, and the two main physical parameters which affect the technology choice and operational costs are the cutting speed and the gas consumption.

#### 2.1.1 Laser cutting speed

**Fig 1:**
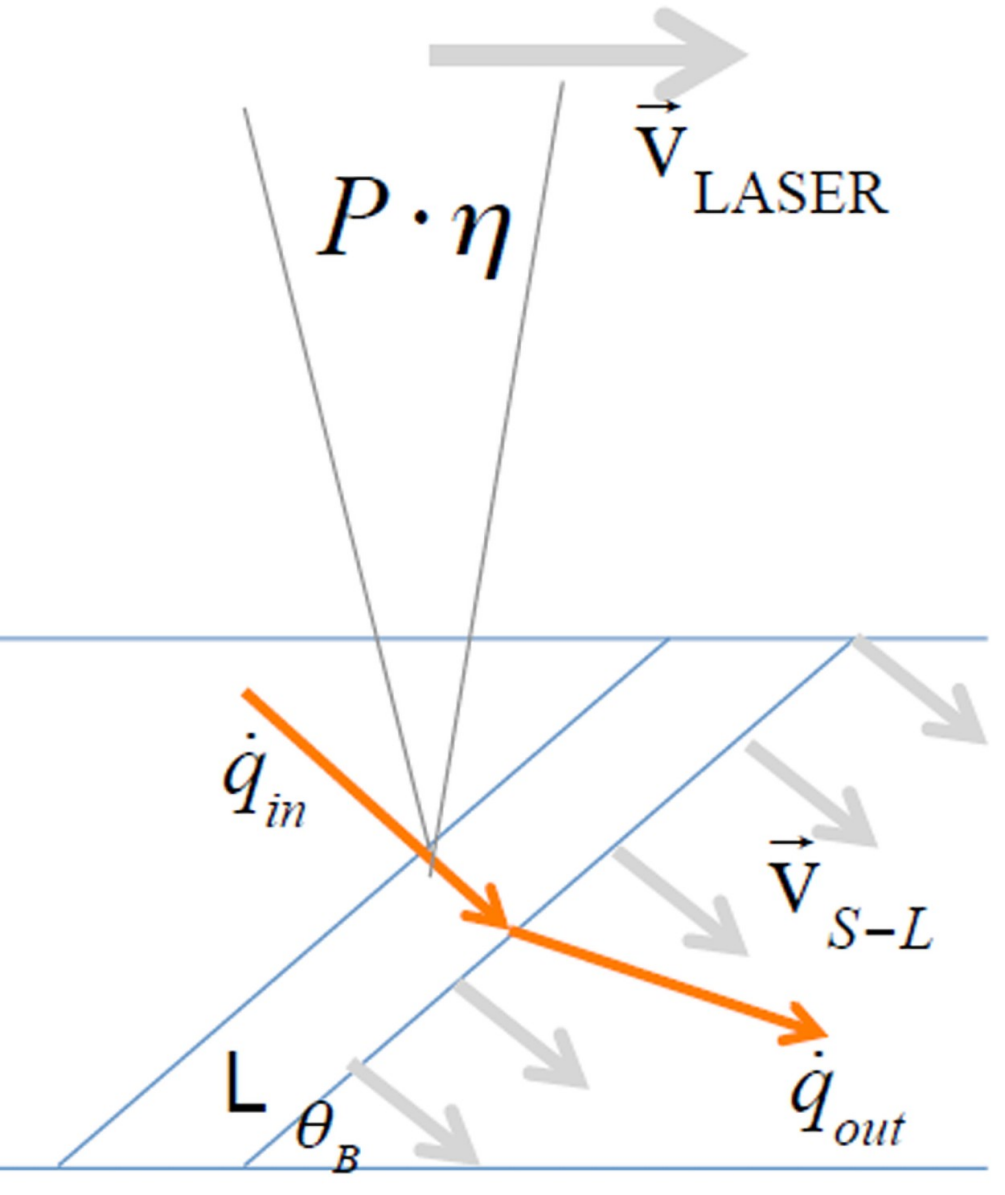
Representation of laser cutting dynamics process in a material.

Laser cutting speed is one of the main physical parameters in these advanced production systems. It controls the heat transfer from the laser beam into the material, and it requires to be adjusted according to the material type to be cut and its thickness. Fig 1 illustrates the laser beam interacting with the material inside the kerf; all the involved parameters in the process are described in Table 1. There is an inverse relationship between cutting velocity and material thickness; hence thick materials need lower cutting speed while thinner ones can be cut at higher cutting speeds. A simple model for understanding the physical process is described as follows.

**Table 1:** Component terms of equation for laser cutting.

*P*_*c*_:	Laser Power, *W*
*w*:	kerf, *m*
*t*:	thickness, *m*
$\vec{v_{c}}$:	cutting speed, *m*/*s*
*ρ*:	density of the processing material, *kg*/*m*^3^
*η*_*c*_:	efficiency of laser beam

Every cutting process, regardless of its physical nature, can be modeled by the first law of thermodynamics [64] as an energy rate or power equation, which relates the volume of the material removed per unit of time ($\dot{V_{c}}$) and the specific energy (*E*_*c*_) involved in the cutting process. Eq 1 represents that relation. If the equation is expanded, then:

$$\begin{array}{c} \eta_{c}\cdot P_{c}=\dot{V_{c}}\cdot E_{c} \end{array}$$ (1)

To develop the specific energy parameter, we combine the latter expression with the Stefan equation, representing the energy balance at the melting interface or cutting front (including the phase change from solid to liquid of the metal); this corresponds to the location where the laser interacts with the surface of the material inside the kerf [59].

$$\begin{array}{c} \dot{m}\Delta H_{S-L}={\dot{q}}_{in}-{\dot{q}}_{out} \end{array}$$ (2)

where $\dot{m}$ represents the mass flow ejected from the cutting front inside the kerf, Δ*H*_*S*−*L*_ represents the phase change of the material as a result of the melting process and $\dot{q}$ corresponds to the flow of heat transferred. Then, from Eq 2 we expand every single term to obtain:

$$\begin{array}{c} {\vec{v}}_{S-L}|\rho A\Delta H_{S-L}=K_{L}A\frac{dT}{dx}{{|}_{L}-K_{S}A\frac{dT}{dx}|}_{S} \end{array}$$ (3)

Here *K*_*L*_ and *K*_*S*_ corresponds to the heat conductivity of the metal in liquid and solid phase, respectively. From Eq 3, the maximum possible velocity is achieved when: $P_{c}\eta_{c}=K_{L}A\frac{dT}{dx}{|}_{L}$ and $\frac{dT}{dx}{|}_{S}=0$. When we replace this condition into Eq 3 we further obtain:

$$\begin{array}{c} |{\vec{v}}_{S-L}|=\frac{\frac{P_{c}\eta_{c}}{A\rho }}{\Delta H_{S-L}}=\frac{P_{c}\cdot \eta_{C}\cdot \text{cos}\theta_{B}}{t\cdot w\cdot \rho \cdot \Delta H_{S-L}} \end{array}$$ (4)

Eq 4 describes the mathematical relation between the parameters and the laser cutting speed for different materials.

**Fig 2:**
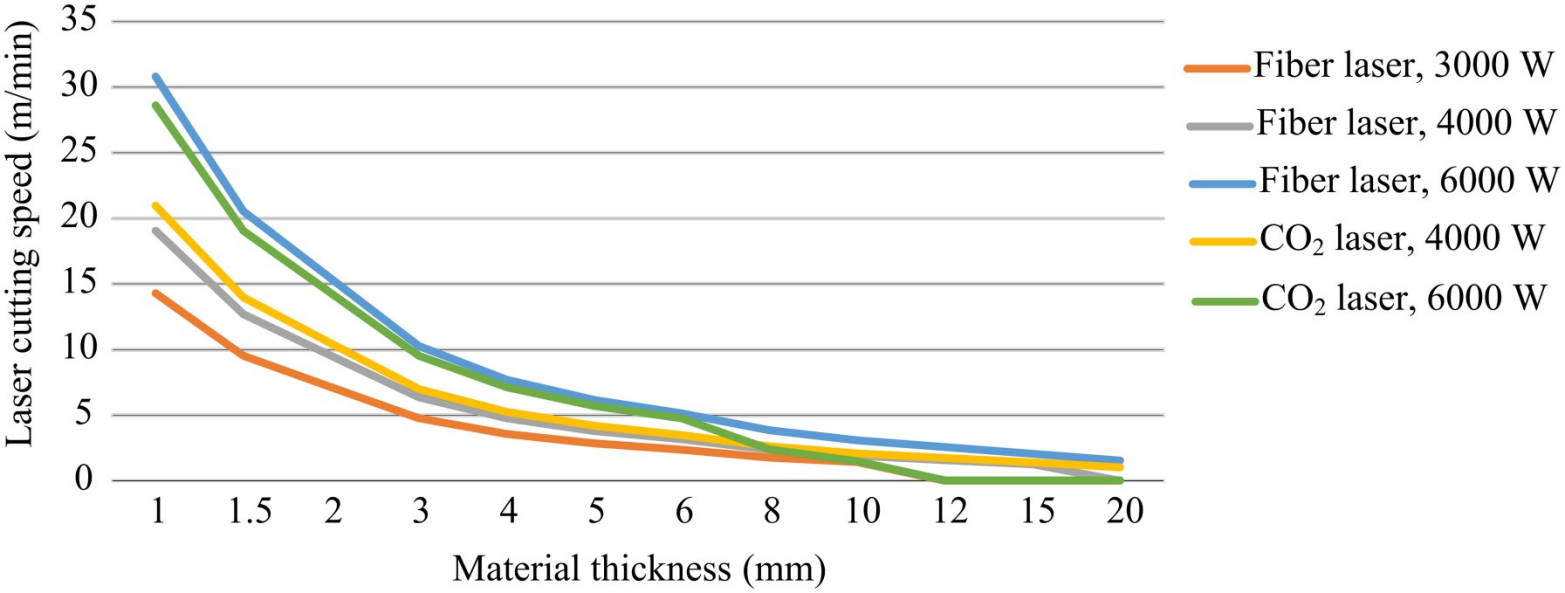
Relationship between laser cutting speed vs thickness for different laser types and power, for steel material.

In the present work, we model two different laser technologies, fiber LASER and *CO*_2_ LASER. Different power values are used for both technologies to obtain the cutting velocity. Table 2 shows the LASER type and the power of each of them, and also the abbreviation that we used in the text. Fig 2 shows the behavior of the laser cutting velocity for both methods and different laser power and thickness of a specific material.

**Table 2:** LASER cutting machines specs.

LASER Type	LASER Power (W)	Tag
Fiber	3,000	A1
Fiber	4,000	A2
Fiber	6,000	A3
*CO*_2_	4,000	B1
*CO*_2_	6,000	B2

#### 2.1.2 Gas consumption model

**Fig 3:**
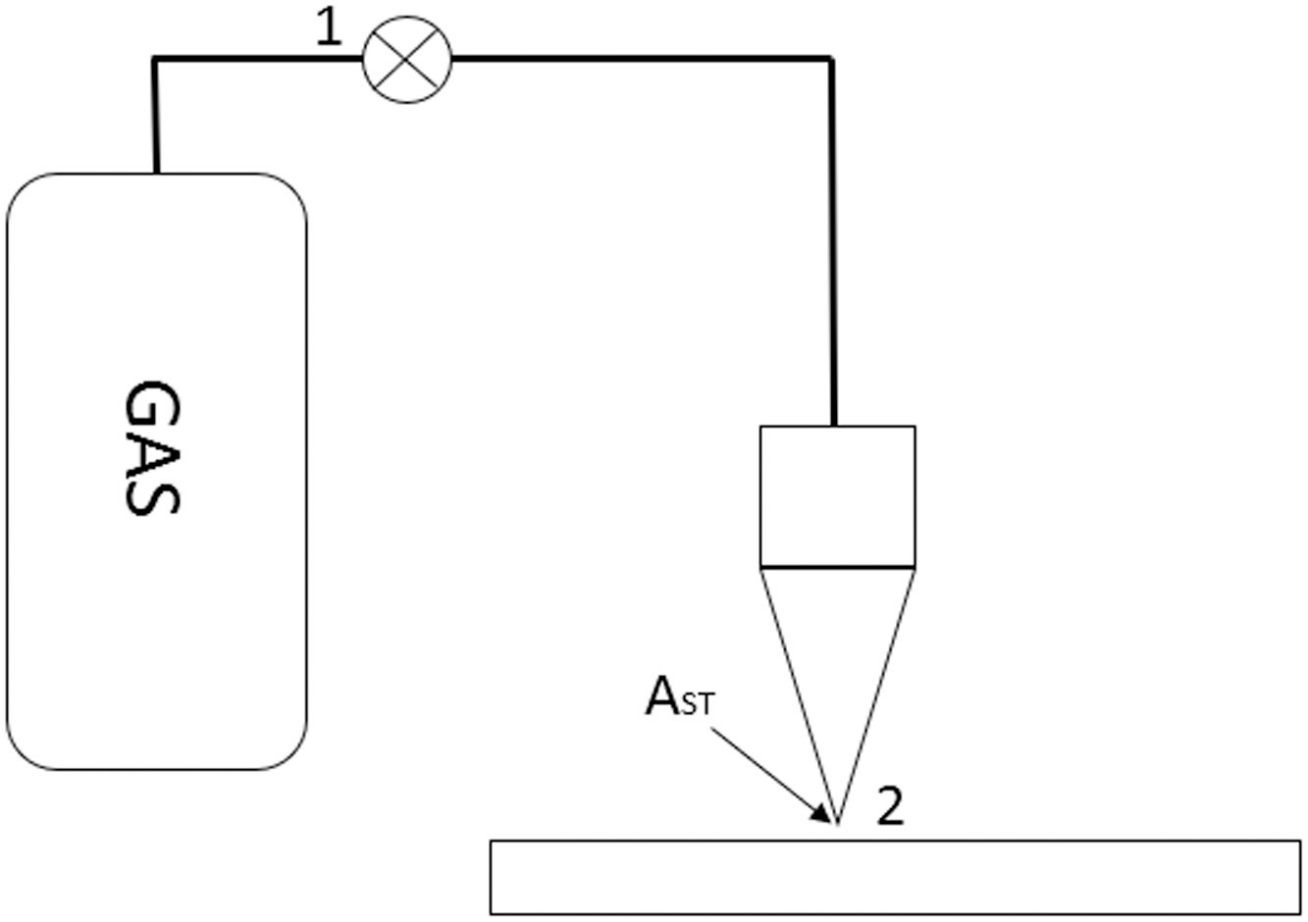
Representation of gas delivery in laser cutting process.

A second important parameter to decide which laser cutting machines technology must be acquired is the amount of gas the machine consumes during the cutting process, since gas consumption is one of the most relevant costs of the laser cutting process operation. Fig 3 shows a schematic of the gas supply device. There is a gas cylinder, a gas regulator (1) and the nozzle (2), through which the coaxial laser beam exists from it. A more extensive explanation of the process is discussed as follows:

First, we assume an incompressible gas, so we can use Bernoulli equation. We evaluate its properties at two points: the gas regulator exit and the nozzle exit. With this approach, we estimate the speed at which the gas exits the nozzle. Then, from the latter the gas flow during the cutting process is determined.

$$\begin{array}{c} \frac{P_{1}}{\rho }+\frac{1}{2}{\vec{v}}_{1}^{2}+z_{1}\cdot g=\frac{P_{2}}{\rho }+\frac{1}{2}{\vec{v}}_{2}^{2}+z_{2}\cdot g \end{array}$$ (5)

Where *P*_*i*_ represents the pressure of the gas on each point in the flow line, *ρ* represents the density of the gas we are modelling, ${\vec{v}}_{i}$ is the speed of the gas at each stage, *z*_*i*_ represent the elevation point with respect to the reference line, and *g* represents the acceleration of gravity. Without losing generality we can assume that *z*_1_ and *z*_2_ are closed enough in magnitude due to the configuration of the system. We can also assume that ${\vec{v}}_{1}$ is near to 0, as at this point the motion of the gas is almost null because *A*_1_ > >*A*_2_. Thus, we obtain:

$$\begin{array}{c} {\vec{v}}_{2}=\sqrt{2(\frac{P_{1}}{\rho_{1}}-\frac{P_{2}}{\rho_{2}})} \end{array}$$ (6)

To be more specific, *P*_1_ is the pressure of the cylinder and *P*_2_ typically corresponds to the atmospheric pressure. However, as gases are compressible in nature, assuming that they behave ideally and that the expansion process is isentropic, we can utilize the isentropic relation for ideal gases under constant-specific-heat assumption [64]

$$\begin{array}{c} {\left(\frac{T_{2}}{T_{1}}\right)}_{s=const}={\left(\frac{P_{2}}{P_{1}}\right)}^{\frac{k-1}{k}} \end{array}$$ (7)

where *s* represent the entropy of the gas, and *k* is the polytropic coefficient obtained as the ratio between the specific heat at constant pressure *c*_*p*_ and the specific heat at constant volume *c*_*v*_. If we isolate *T*_2_ we obtain the following isoentropic exit temperature relationship:

$$\begin{array}{c} T_{2}=T_{1}{\left(\frac{P_{2}}{P_{1}}\right)}^{\frac{k-1}{k}} \end{array}$$ (8)

From the latter expression, we can estimate the sound velocity of the gas at that same point using the kinetic theory of gases which is represented by Eq 9. Here, *γ*_*i*_ represents the adiabatic gas constant, *R*_*gas*_ is the ideal gas constant and *T*_*i*_ is the temperature at the exit of the nozzle.

$$\begin{array}{c} {\vec{v}}_{i}=\sqrt{\gamma_{i}\cdot R_{gas}\cdot T_{i}} \end{array}$$ (9)

If the gas velocity calculated using Eq 6 becomes larger in magnitude than the sound velocity given by Eq 9. Then, the gas pressure at point 1 must be lowered to avoid the compressibility effect of the gas when using a convergent nozzle. This effect tends to choke the nozzle exit and dissipate a considerable amount of gas flow energy; reducing the available momentum in the laser-material interaction cutting zone to remove the molten dross and thus diminishing the quality of final cut. The latter problem can be fixed using a convergent-divergent or De Laval nozzle, however that consideration falls outside the scope of this research. Finally, in order to obtain the gas flow through the nozzle, *Q*, we multiply the exist gas velocity by the cross section area of the nozzle.

$$\begin{array}{c} Q={\vec{v}}_{2}\cdot A_{nozzle} \end{array}$$ (10)

It is important to take into consideration that each material-thickness combination requires a specific nozzle exit diameter value.

### 2.2 Deterministic optimization model formulation

Using the previous developed equations of laser speed and gas consumption for each type of technology we will proceed now to present the cost optimization model to determine the optimal equipment selection and production planning for a AMT laser cutting company. We will first present the model indexes, variables and parameters and then the formulation of the deterministic optimization model will be depicted.

$$\begin{array}{c} \begin{array}{cc} \text{(Q1)}\;\text{Max}\;z= & \sum_{t}[\sum_{p}\sum_{m}\sum_{j}\frac{x_{p,t}^{m,j}}{\nu_{p}^{m}}\left(\alpha_{p}-\left(G_{p}^{m}+E_{p}^{m}\right)\right)\\ & -\sum_{p}\rho_{p}\left(D_{p,t}-\sum_{m}\sum_{j}\frac{x_{p,t}^{m,j}}{\nu_{p}^{m}}\right)-\sum_{m}\sum_{j}y_{t}^{m,j}\gamma^{m}]\frac{1}{{\left(1+r\right)}^{t}} \end{array} \end{array}$$ (11)

subject to:

$$\begin{array}{c} \sum^{M}\sum^{J}\frac{x_{p,t}^{m,j}}{\nu_{p}^{m}}\leq D_{p,t}\;\forall p,t \end{array}$$ (12)

$$\begin{array}{c} \sum^{M}y_{t}^{m,j}\leq 1\;\forall t \end{array}$$ (13)

$$\begin{array}{c} \sum^{P}\sum^{M}\sum^{J}\frac{x_{p,t}^{m,j}}{\nu_{p}^{m}}\leq \Omega \sum^{M}\sum^{J}w_{t}^{m,j}\;\forall t \end{array}$$ (14)

$$\begin{array}{c} x_{p,t}^{m,j}\leq Mw_{t}^{m,j}\;\forall p,m,j,t \end{array}$$ (15)

$$\begin{array}{c} y_{t-1}^{m,j}=w_{t}^{m,j}-w_{t-1}^{m,j}\;\forall m,j,t \end{array}$$ (16)

$$\begin{array}{c} x_{p,t}^{m,j}=0\;\forall i,j,t\notin Mat^{m} \end{array}$$ (17)

$$\begin{array}{c} w_{0}^{m,j}=1\;\forall j\notin Maq^{m} \end{array}$$ (18)

$$\begin{array}{c} y^{m,t},w^{m,t}\in \left(0,1\right)\;|\forall m,t \end{array}$$ (19)

$$\begin{array}{c} x_{p,t}^{m,j}\geq 0\;|\forall p,t,m,j \end{array}$$ (20)

The equipment selection and production planning problem (Q1) can be modeled as a linear programming optimization model. In Table 3 we can observe the indexes of the model, Table 4 presents the variables of the model and finally Table 5 shows the parameters of the model. At this stage the model is deterministic, so we do not consider uncertainty. Later we will present how to add uncertainty into the model by applying robust optimization.

**Table 3:** Indexes.

P:	Set of Products (indexed by *p*)
M:	Set of Machines (indexed by *m*)
T:	Set of Periods (indexed by *t, j*)

**Table 4:** Variables.

$x_{p,t}^{m,j}$:	Cutting length of product *p*, in machine *m,j* in period *t*
$w_{t}^{m,j}$:	1 if a machine *m,j* is available on period *t*, 0 if not
$y_{t}^{m,j}$:	1 if an investment decision on machine *m,j* is made on period *t*, 0 if not

**Table 5:** Parameters.

*α*_*p*_:	sale price for each cutting minute of product *p*
$\nu_{p}^{m}$:	cutting speed for each product *p* in machine *m*
$G_{p}^{m}$:	sale cost for each minute of gas consumed by machine *m* in product *p*
$E_{p}^{m}$:	sale cost for each minute of electricity consumed by machine *m* in product *p*
*D*_*p*,*t*_:	Demand of product *p* in period period *t*
*ρ*_*p*_:	penalty cost for each minute of incomplete demand of product *p*
*γ*^*m*^:	Price of machine *m*
Ω:	Available time for each period
*r*:	Discount rate
*Mat*^*m*^:	set of products that machine *m* can process
*Maq*^*m*^:	set of machines that can not be acquired

The objective function (Q1) maximizes the company profit by obtaining the operational income of each type of technology and subtracts both the investment cost and the cost of lost sales. We determine the operational cost for each product and machinery combination, by determining the equivalent cutting speed (calculated in section 4.2.1) and relate it to the time required to cut and finally obtaining the gas and electricity consumption during the process. The cost of not filling the demand was estimated as 30% of the selling price of each product, this value is related to the lost revenue due to the unfilled demand. The first constraint (see Eq 12 ensures that on each period of time, production time sold for a specific item is never higher than the demand. Constraint 13 guarantees that the company can only buy one machine per period of time, due to the considerable cost of each piece of equipment. Constraint 14 limits the use of the machinery to the available operational time during each period. We define an operational time as 145,800 minutes per machine per period. Constraint 15 represents the relation between two decision variables, where a machine is unavailable for production if it has not been purchased. Constraint 16 shows that each machine can only process specific products. Constraints 17 and 18 represent the initial condition of the problem. Finally, constraints 19 and 20 correspond to the nature of each decision variable.

## 3 Robust reformulation

In this section, we will describe how uncertainty was added into the previous formulation using a RO approach proposed by [49]. First, we will describe how the RO methodology works and then apply it to model the demand uncertainty.

### 3.1 Robust optimization methodology

Our first assumption is that uncertainty in the parameters affects only the elements in the right hand of the constraint matrix. We will start with a general representation of the deterministic model, given by:

$$\begin{array}{c} \;\text{minimize}\;c^{T}x \end{array}$$

$$\begin{array}{c} \text{subject}\;\text{to:}\;a_{i}^{T}\leq b_{i}\;\forall i=1,...,m \end{array}$$

$$\begin{array}{c} \;l\leq x\leq u \end{array}$$ (21)

To model uncertainty into the constraints (**A**), each uncertain coefficient *a*_*i*,*j*_ can take values based on a symmetric distribution with mean equal to the nominal value *a*_*i*,*j*_ in the interval $\left[{\bar{a}}_{i,j}-{\hat{a}}_{i,j},{\bar{a}}_{i,j}+{\hat{a}}_{i,j}\right]$. The exact value of the deviation component ${\hat{a}}_{i,j}$ is unknown, but can be estimated. As is unlikely that all coefficients will be equal to their nominal value, it is also unlikely that they will be equal to their worst-case value.

The objective now is to adjust the level of conservatism of the solution, in order to achieve a reasonable trade-off between robustness and performance. On literature, authors defined the scaled deviation of a parameter *a*_*i*,*j*_ from its nominal values as $z_{i,j}=\left(a_{i,j}-{\bar{a}}_{i,j}\right)/{\hat{a}}_{i,j}$. This variable can only take values between [-1,1]. For each constraint that is subject to uncertainty, a threshold (not necessarily integer) is introduced to bound the total variations of uncertain parameters, as follows:

$$\begin{array}{c} \sum_{(i,j)\in J}\left|z_{i,j}\right|\leq \Gamma \end{array}$$

where *J* is the set of indexes of uncertain parameters. When Γ = 0 we obtain a average case, and when it takes Γ = |*J*| we get the worst case. Bertsimas & Sim [49] states that this allows greater flexibility to build a robust model without excessively affecting the optimum of the optimization problem. This decision will be defined according to the risk aversion of the decision maker. To build the robust counterpart of the nominal problem, we will follow the uncertainty set proposed by [49]. It is constructed by maximizing the left-hand side of the constraints over the set of admissible scaled deviations. This leads to the following problem:

$$\begin{array}{c} \;\text{maximize}\;c^{T}x \end{array}$$

$$\begin{array}{c} \;\text{subject}\;\text{to:}\;{\bar{a}}_{i}^{T}x+\beta_{i}\left(x,\Gamma_{i}\right)\leq b_{i},\;\forall i=1,...,m \end{array}$$

$$\begin{array}{c} \;x\geq 0 \end{array}$$ (22)

where ${\bar{a}}_{i}^{T}$ represents the nominal data for row *i* and the protection function for each constraint *i* = 1, …, *m* is defined as a new optimization problem, which objective function consist in: $\beta_{i}\left(x,\Gamma_{i}\right)=\text{maximize}\sum_{j\in J_{i}}\left|x_{j}\right|{\hat{a}}_{i,j}z_{i,j}$ subject to $\sum_{j\in J_{i}}\leq \Gamma_{i}$ and ≤ *z*_*i*,*j*_ ≤ 1, ∀*j* ∈ *J*_*i*_. By the application of strong duality we can reformulate the problem as equivalent to:

$$\begin{array}{c} \text{maximize}\;c^{T}x \end{array}$$

$$\begin{array}{c} \text{subject}\;\text{to:}\;{\bar{a}}_{i}^{T}x+z_{i}\Gamma_{i}+\sum_{j\in J_{i}}p_{i,j}\leq b_{i},\;\forall i \end{array}$$

$$\begin{array}{c} z_{i}+p_{i,j}\geq {\hat{a}}_{i,j}y_{j},\;\forall i,j\in J_{i} \end{array}$$

$$\begin{array}{c} -y_{j}\leq x_{j}\leq y_{j},\;\forall j \end{array}$$

$$\begin{array}{c} y_{j},z_{i},x_{j}\geq 0,\;\forall j \end{array}$$

$$\begin{array}{c} p_{i,j}\geq 0,\;\forall i,j\in J_{i} \end{array}$$ (23)

where variables *z*_*i*_ and *p*_*i*,*j*_ are dual variables of the problem 23. The problem is linear, so there is no difficulty in solving using standard linear methods.

### 3.2 Uncertainty in demand

We will consider uncertainty only on the demand parameter *D*_*p*,*t*_. Using a RO approach we will find solutions, which up to a certain level, are still feasible up to certain degree of demand variability.

To simplify the modeling of the robust counterpart of the model (Q1), we will substitute *D*_*p*,*t*_ *ρ*_*p*_ with Π_*p*,*t*_ in the objective function, and add the following constraint to the model.

$$\begin{array}{c} D_{p,t}\rho_{p}\geq \Pi_{p,t}\;|\forall p,t \end{array}$$ (24)

This change of variable allows us to remove *D*_*p*,*t*_ *ρ*_*p*_ from the objective function without affecting the problem’s solution. To re-formulate (Q1) as a robust model, we will rewrite constraints 12 and 24 which contain an uncertain parameter and add Γ_*p*,*t*_ as the uncertainty budget for the new constraints.

$$\begin{array}{c} \beta_{p,t}\left({\hat{D}}_{p,t};\Gamma_{p,t}\right)=\text{Max}\;\left\{\sum_{l}^{t}\rho_{p}z_{p,l}{\hat{D}}_{p,l}\right\} \end{array}$$

$$\begin{array}{c} \text{subject}\;\text{to:}\;\sum_{l}^{t}z_{p,l}\leq \Gamma_{p,t}\;\forall p,l\leq t \end{array}$$

$$\begin{array}{c} 0\leq z_{p,l}\leq 1\;\forall p,l\leq t \end{array}$$ (25)

Using duality we can transform 25 into the following problem:

$$\begin{array}{c} \text{(Q2)}\;\text{min}\;\{v_{p,t}\Gamma_{p,t}+\sum_{l}^{t}s_{l,p,t}\} \end{array}$$

$$\begin{array}{c} \text{subject}\;\text{to:}\;v_{p,t}+s_{l,p,t}\geq {\hat{D}}_{l,k}\;|\forall p,t,l\leq t \end{array}$$ (26)

$$\begin{array}{c} v_{p,t},s_{l,p,t}\geq 0\;|\forall l\leq t \end{array}$$ (27)

We can write another protection function for constraint 12 of the deterministic model. If we replace the original deterministic model constraints with the robust ones, by the previous obtained protection functions, we obtain:

$$\begin{array}{c} \sum_{j}^{T}\sum_{m}^{M}\frac{x_{p,t}^{m,j}}{\nu_{p}^{m}}\leq {\bar{D}}_{p,t}-\Gamma_{p,t}q_{p,t}-\sum_{k}^{t}u_{k,p,t}\;|\forall p,t,k\leq t \end{array}$$ (28)

$$\begin{array}{c} \Pi_{p,t}\geq {\bar{D}}_{p,t}+\Gamma_{p,t}v_{p,t}+\sum_{l}^{t}s_{l,p,t}\;|\forall p,t,l\leq t \end{array}$$ (29)

Additionally, we add two more constraints to the original problem to account for the relation between dual variables, the uncertainty budget *v*_*p*,*t*_, *q*_*p*,*t*_, *s*_*l*,*p*,*t*_, *u*_*k*,*p*,*t*_ and uncertainty variable (constraints 26 and 28). We also add constraints corresponding to the nature of the new variables (27 and 29).

The full robust counterpart (Q3) is the following:

$$\begin{array}{c} \begin{array}{cc} \text{(Q3)}\;\text{Max}\;\text{z}=\sum_{t} & [\sum_{p}\sum_{m}\sum_{j}\frac{x_{p,t}^{m,j}}{\nu_{p}^{m}}\left(\alpha_{p}+\rho_{p}-\left(G_{p}^{m}+E_{p}^{m}\right)\right)-\\ & \sum_{p}\rho_{p}\Pi_{p,t}-\sum_{m}\sum_{j}y_{t}^{m,j}\gamma^{m}]\frac{1}{{\left(1+r\right)}^{t}} \end{array} \end{array}$$ (30)

subject to:

$$\begin{array}{c} \sum_{j}^{T}\sum_{m}^{M}\frac{x_{p,t}^{m,j}}{\nu_{p}^{m}}\leq {\bar{D}}_{p,t}+\Gamma_{p,t}q_{p,t}+\sum_{k}^{t}u_{k,p,t}\;|\forall p,t,k\leq t \end{array}$$ (31)

$$\begin{array}{c} \Pi_{p,t}\geq {\bar{D}}_{p,t}+\Gamma_{p,t}v_{p,t}+\sum_{l}^{t}s_{l,p,t}\;|\forall p,t,l\leq t \end{array}$$ (32)

$$\begin{array}{c} v_{p,t}+s_{l,p,t}\geq {\hat{D}}_{l,k}\;|\forall p,t,l\leq t \end{array}$$ (33)

$$\begin{array}{c} q_{p,t}+u_{k,p,t}\geq {\hat{D}}_{p,k}\;|\forall p,t,k\leq t \end{array}$$ (34)

$$\begin{array}{c} \sum^{M}y_{t}^{m,j}\leq 1\;\forall t \end{array}$$ (35)

$$\begin{array}{c} \sum^{P}\sum^{M}\sum^{J}\frac{x_{p,t}^{m,j}}{\nu_{p}^{m}}\leq \Omega \sum^{M}\sum^{J}w_{t}^{m,j}\;\forall t \end{array}$$ (36)

$$\begin{array}{c} x_{p,t}^{m,j}\leq Mw_{t}^{m,j}\;\forall p,m,j,t \end{array}$$ (37)

$$\begin{array}{c} y_{t-1}^{m,j}=w_{t}^{m,j}-w_{t-1}^{m,j}\;\forall m,j,t \end{array}$$ (38)

$$\begin{array}{c} x_{p,t}^{m,j}=0\;\forall i,j,t\notin Mat^{m} \end{array}$$ (39)

$$\begin{array}{c} w_{0}^{m,j}=1\;\forall j\notin Maq^{m} \end{array}$$ (40)

$$\begin{array}{c} y^{m,t},w^{m,t}\in \left(0,1\right)\;|\forall m,t \end{array}$$ (41)

$$\begin{array}{c} x_{p,t}^{m,j},q_{p,t},u_{l,p,t},v_{p,t},s_{k,p,t}\geq 0\;|\forall p,t,m,j,k,l \end{array}$$ (42)

The objective function of (Q3) is similar to the last one (Q1). The main difference is the term Π_*p*,*t*_ which represents the uncertain parameter (demand). Most constraints equal the ones of (Q1) [35–41]. The new demand satisfaction constraint is 31, which has the nominal value of the demand, the uncertainty budget and the dual variables of the problem (Q2). Constraint 32 allows the removal of the demand from the objective function. This constraint has the nominal value of the uncertain parameter, the uncertainty budget and the dual variables of the problem (Q2). Constraints 33 and 34 are related with the transformation of the linear problem into the robust problem through the hard duality theorem of optimization.

## 4 Numerical case study

**Fig 4:**
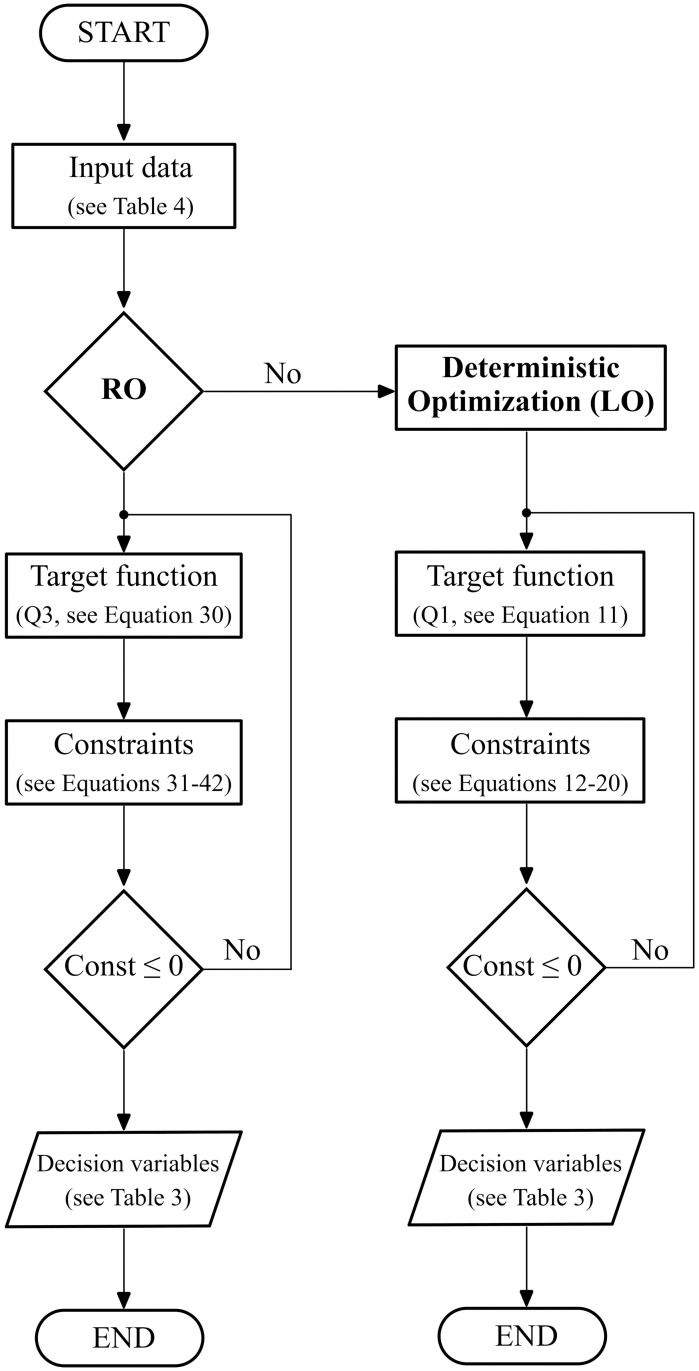
Flow chart of the optimization process.

In this section, we will apply our models and perform a numerical case study using the values of a company which offers laser cutting services. The model will determine which should be the best configuration of machinery and production scheme for given level of demand uncertainty. We will determine the technology decision, from a given pool of options available in the market, and amount of machines that should be acquired and the production plan needed to fill out the demand. To better understand the whole procedure carried out to solve the case of study a flow chart is added (see Fig 4). For this study, we will have available five types of laser beam cutting technologies according to the nominal power that they use. According to the nomenclature proposed, letters A and B correspond to fiber and *CO*_2_ lasers respectively. Table 6 presents the information for each type of technology. Additionally, we consider that 11 different types products (*P*) to be processed. Not all technologies can cut all products, so in Table 7 we present which products can be processed on each machine. The model is executed for six periods of time (*T*) which is equivalent to 5 years. The discount rate is *σ* = 0.1. The companies starts with two types of technologies: A2 and B1. We assume a normal distribution for the demand of each product, where the mean and standard deviation are based on historical information.

**Table 6:** Available types of technology.

Laser type	Machine type and technology				
	A1	A2	A3	B1	B2
A	X	X	X		
B				X	X

**Table 7:** Types of products which can be process on each machine technology.

Equipment	P1	P2	P3	P4	P5	P6	P7	P8	P9	P10	P11
A1	X	X		X	X	X	X	X	X		X
A2	X	X	X	X	X	X	X	X	X		X
A3	X	X	X	X	X	X	X	X	X		X
B1	X	X	X	X	X		X	X	X	X	
B2	X	X	X	X	X		X	X	X	X	

We programmed the optimization models using the IBM ILOG CPLEX Optimization Studio (64 bit) V12.6.1.0, and there were optimized using a computer with 8 GB of RAM and a 2.4GHz dual-core Intel Core i7.

Laser cutting and gas consumption parameters model were determined for each technology using the previous equations. These numbers are presented in Tables 8 and 9. Table 10 presents the ratio between laser cutting speed and the equipment price. Demand was estimated from the company that offers laser cutting services, using historical information between 2012 and 2017. Demand information, expressed as a percentage of demand for each special products over the total demand for each year, can be observed in Table 11

**Table 8:** Gas consumption levels for each technology obtained from Eq 10.

P_*p*_	Q *m*^3^/*hr*	$/hr
P1	0.9117	147.6
P2	0.8754	151.2
P3	0.8009	160.2
P4	0.9794	263.4
P5	0.9400	285.0
P6	0.8594	286.8
P7	0.9794	263.4
P8	0.9400	285.0
P9	0.9648	286.8
P10	0.9117	147.6
P11	0.9308	258.0

**Table 9:** Laser cutting speeds for each technology obtained from Eq 4 in m/min.

	Machine type and technology				
P_*p*_	A1	A2	A3	B1	B2
P1	7.15	9.53	15.39	10.48	14.30
P2	2.38	3.18	5.13	3.49	4.77
P3	-	1.59	2.57	1.75	0.04
P4	6.78	9.04	16.68	9.94	13.56
P5	2.26	3.01	3.48	3.31	4.52
P6	1.36	1.81	1.25	1.99	2.71
P7	25.72	34.29	55.39	37.72	51.53
P8	12.86	17.14	27.70	18.86	25.72
P9	8.57	11.43	18.46	12.57	17.14
P10	-	-	-	6.81	9.81
P11	2.38	3.18	5.13	-	-

**Table 10:** Ratio between laser cutting speed and the equipment price, in m/min/$.

	Machines type and technology				
P_*p*_	A1	A2	A3	B1	B2
P1	1.02E-5	1.06E-5	2.23E-5	1.33E-5	1.43E-5
P2	3.40E-6	3.53E-6	7.43E-6	4.42E-6	4.77E-6
P3	-	1.77E-6	3.72E-6	2.22E-6	4.00E-8
P4	9.69E-6	1.00E-5	2.42E-5	1.26E-5	1.36E-5
P5	3.23E-6	3.34E-6	5.04E-6	4.19E-6	4.52E-6
P6	1.94E-6	2.01E-6	1.81E-6	2.52E-6	2.71E-6
P7	3.87E-5	3.81E-5	8.03E-5	4.77E-5	5.15E-5
P8	1.84E-5	1.90E-5	4.01E-5	2.39E-5	2.57E-5
P9	1.22E-5	1.27E-5	2.68E-5	1.59E-5	1.71E-5
P10	-	-	-	8.62E-6	9.81E-6
P11	3.40E-6	3.53E-6	7.43E-6	-	-
Average	9.02E-2	9.52E-6	1.99E-5	1.23E-5	1.31E-5

**Table 11:** Percentage of demand of special products over the total demand per years, in %.

Products	Years					
	0	1	2	3	4	5
P3	23.26	11.82	8.47	6.81	5.80	5.10
P10	0.10	0.17	0.05	0.02	0.06	0.04
P11	0.53	0.26	0.16	0.11	0.09	0.07

## 5 Results and discussion

**Fig 5:**
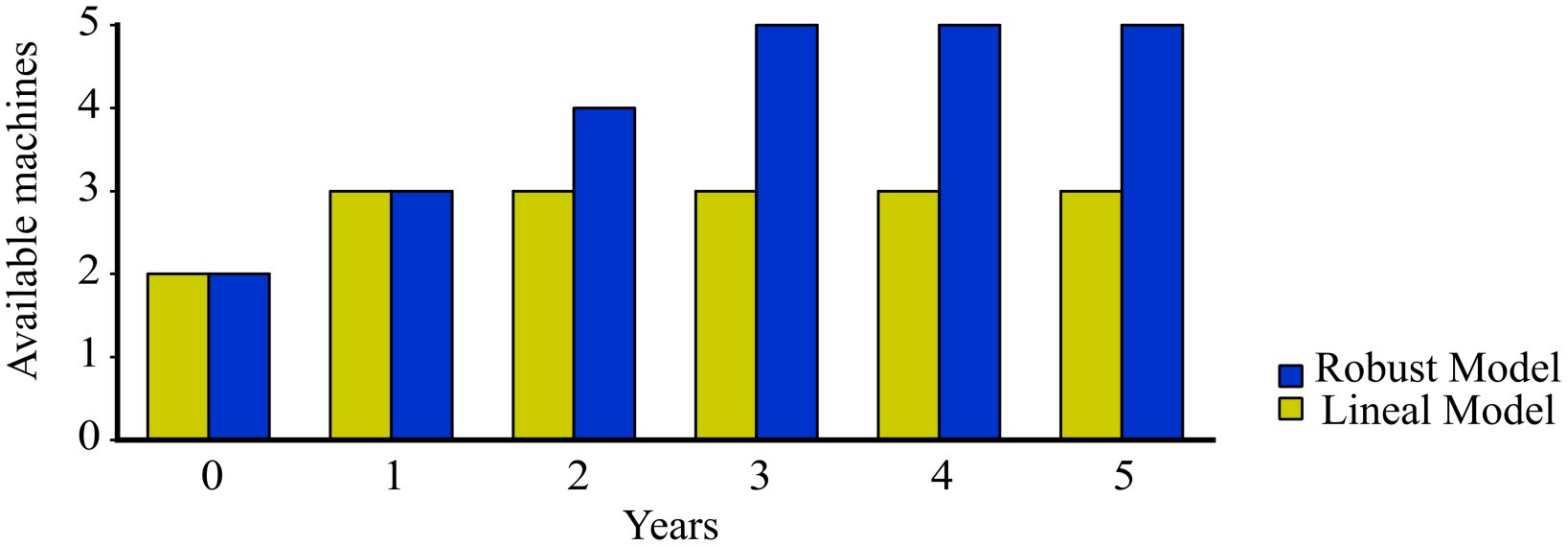
Machinery configuration of the proposed models.

**Fig 6:**
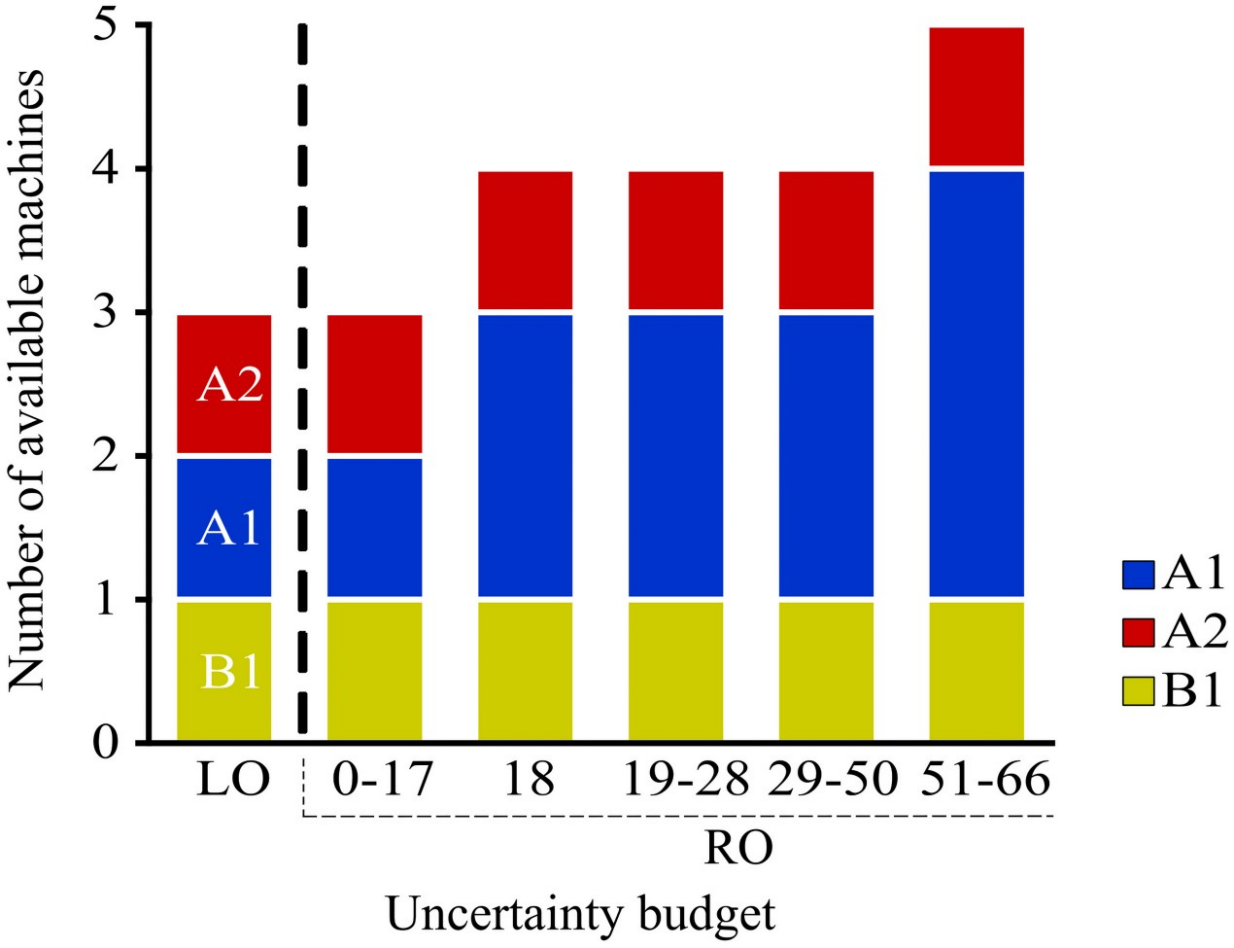
Investment plan for each level of uncertainty LO and RO models.

In this section, we present the results obtained from the deterministic linear optimization (LO) model and the robust optimization (RO) model. In addition, we study the differences that arises between the models and how does the addition of uncertainty in a RO model generates economic benefits. Both models have different results as the RO model considers uncertainty on the demand while the deterministic model does not consider it. In Fig 5 we can observe the results regarding the number of machines that each model proposes for each period. The deterministic model invests in one machine type A1 in the first period, and never invest again. On the other hand, the robust optimization model shows that the optimal decision is to invest on three machines of type A1 in periods 1, 2 and 3. Table 12 and Fig 6 shows that as we increase the uncertainty budget, the investment plan proposed by the robust model involves purchasing additional machines. As the robustness level is increased to a level of 51-66, the model invests each year in an additional machine. The machine with technology type A1 seems to be the preferred one.

**Table 12:** Machinery investment during the years.

Robustness level	0	1	2	3	4	5	Total Machines
LO	A1						3
0-17	A1						3
18	A1			A1			4
19-28	A1		A1				4
29-50	A1	A1					4
51-66	A1	A1	A1				5

**Fig 7:**
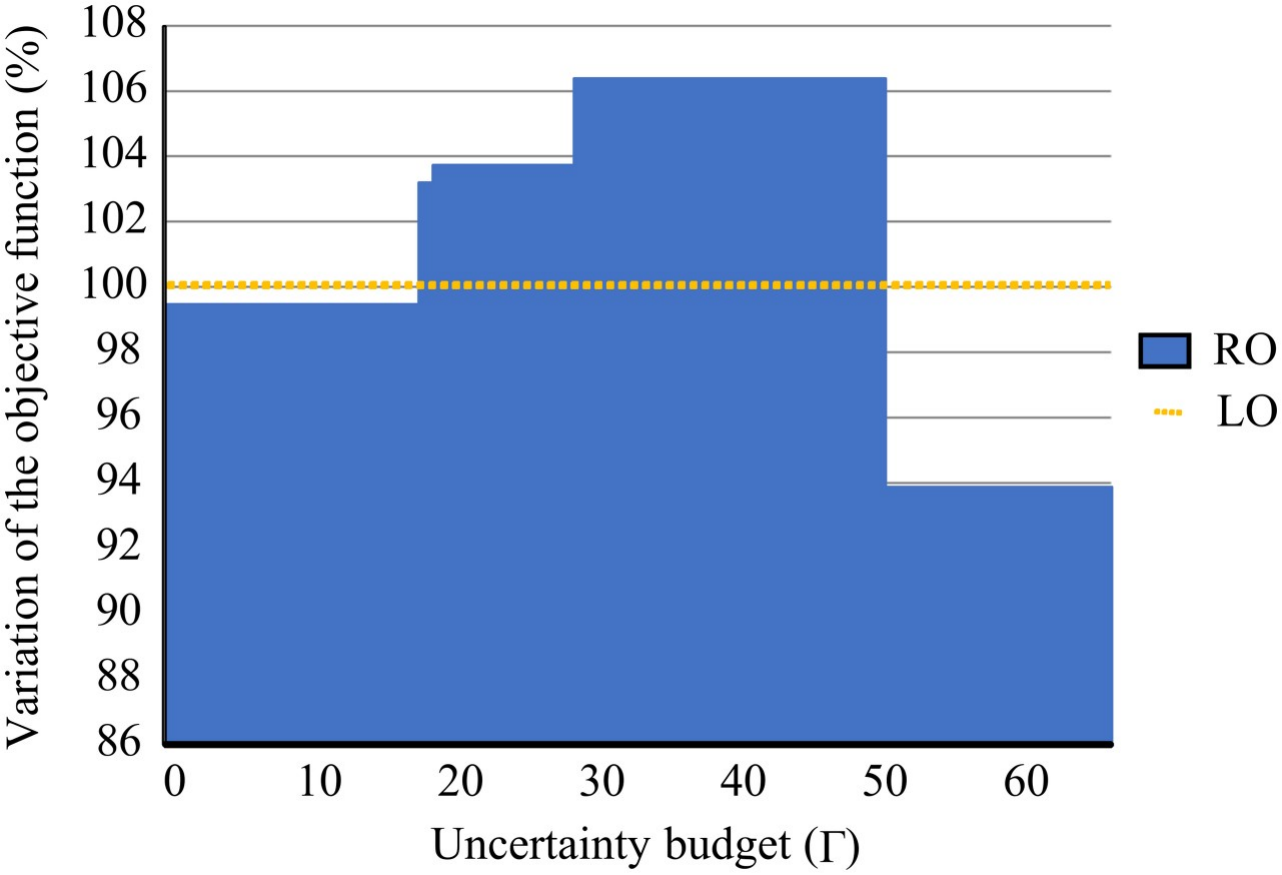
Objective function percentage value for different level of uncertainty budgets.

Fig 7 and Table 13 shows the percentage change and values in the objective function for different uncertainty budgets for a given demand variability level. The optimal production plan in the deterministic model does not include uncertainty, so its value remains stable (yellow dotted line). When the uncertainty budget is less than 17, the result of the RO model is slightly smaller than the result of the deterministic (LO) model because the uncertainty budget is too small to take care of the demand variability. As we increase the uncertainty budget, the RO model surpasses the deterministic model because the technology and productive decisions account for the variability on the demand. When the uncertainty budget reaches the 51-66 level, the objective value drops drastically, below the deterministic model, because the decision maker has over-invested and has created a buffer over the demand uncertainty.

**Table 13:** Objective function value and its change under different levels of uncertainty.

Lineal	Robust	Uncertainty budget	%
4,142,026	4,120,197	0-17	-0.52%
4,142,026	4,274,466	18	3.19%
4,142,026	4,296,642	19-28	3.73%
4,142,026	4,406,702	29-50	6,39%
4,142,026	3,888,294	51-66	-6.13%

**Fig 8:**
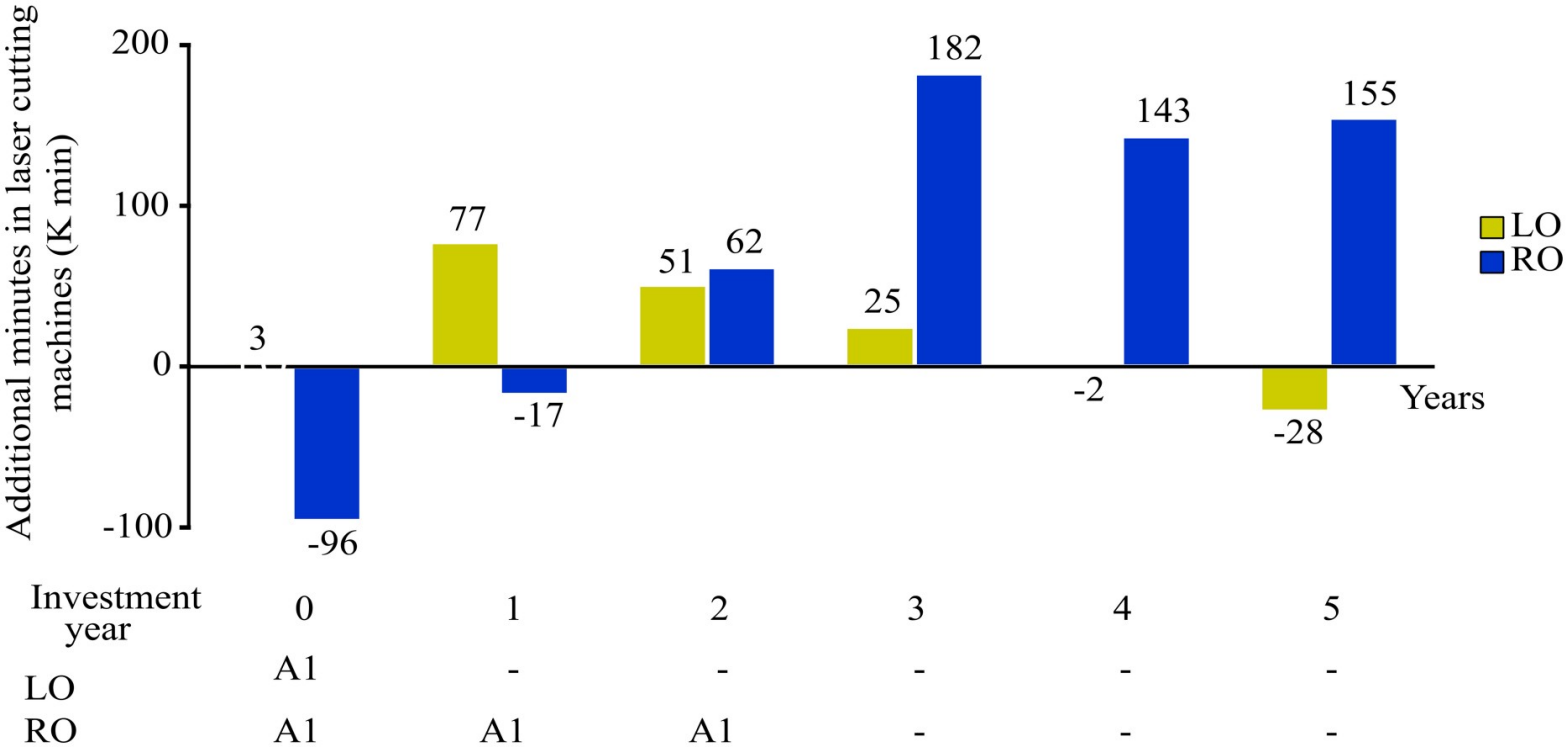
Additional minutes for LO, RO productive plan.

To analyze the effect that the uncertainty budget has on the technology and the productive plan, we must look at the levels of unfilled demand for each product during the considered time frame. Fig 8 show the minutes consumed by the machines during the years. The deterministic (LO) model (i.e., fixed demand) has negative minutes on years 4 and 5 because is unable to fulfill the demand. On the other hand, the RO model during the first two years is unable to fill all the required demand, but during the next years, it invests in more technology having enough capacity to fulfill the required demand.

**Fig 9:**
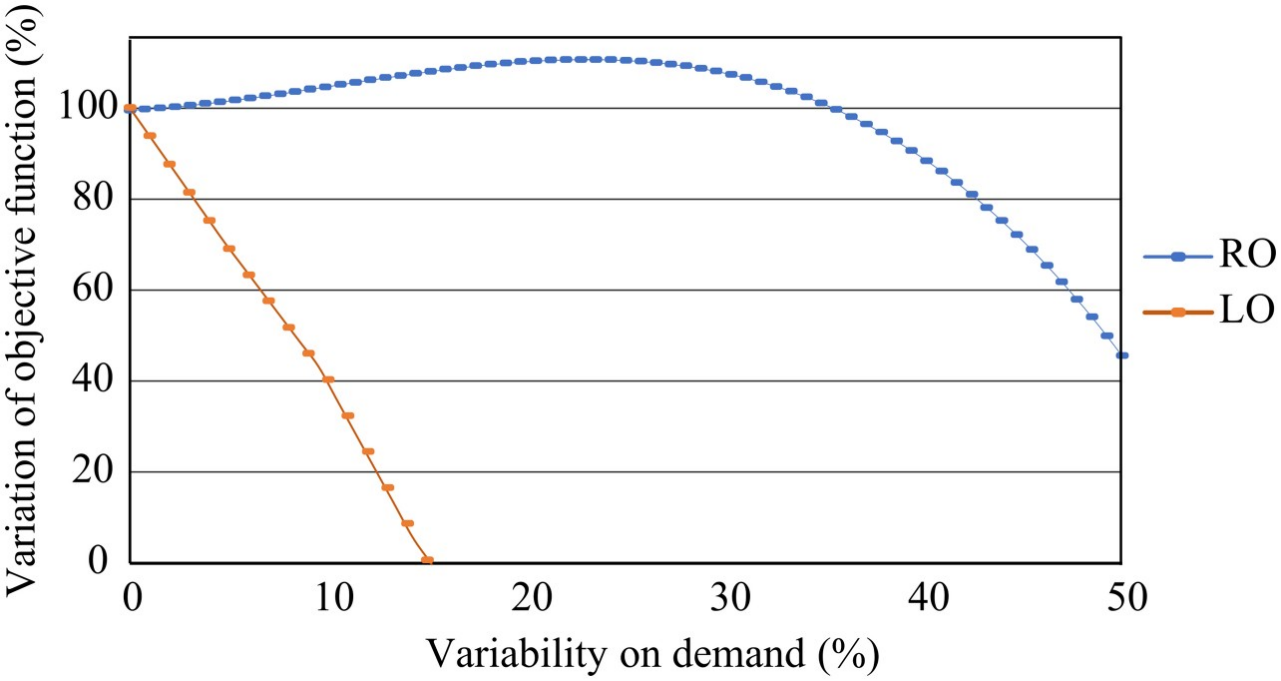
Objective function percentage value for different level of demand uncertainty for a given budget.

The objective behind the robustness budget is to add additional production capacity into the system to be able to take care of the demand variability. Nevertheless this additional capacity is associated to an added cost, hence it is important to determine the adequate level of robustness to cope with the demand variability. Fig 9 and Table 14 shows the objective value under different levels of demand uncertainty for the deterministic (LO) and RO model. For the LO model we can observe that as the demand uncertainty increases the objective value monotonously decreases, indicating that the demand uncertainty negatively affects the deterministic model. On the other hand, if we observe the RO model, as the variability in demand is increased the model performance is improved, because the robustness investment is reflected in a given technology acquisition and production plan which allows the company to adjust better to the demand variability. This improvement reaches a maximum, around a 30% variability, after which it decreases indicating that the robustness is not enough to support the increased level of demand variability.

**Table 14:** Variation of the objective function when demand deviation value changes from 0-50%.

Γ	RO%
0	99.5
1	99.7
2	99.9
3	100.2
:	
10	103.6
20	107.9
30	110.6
40	107.4
50	46.8

**Fig 10:**
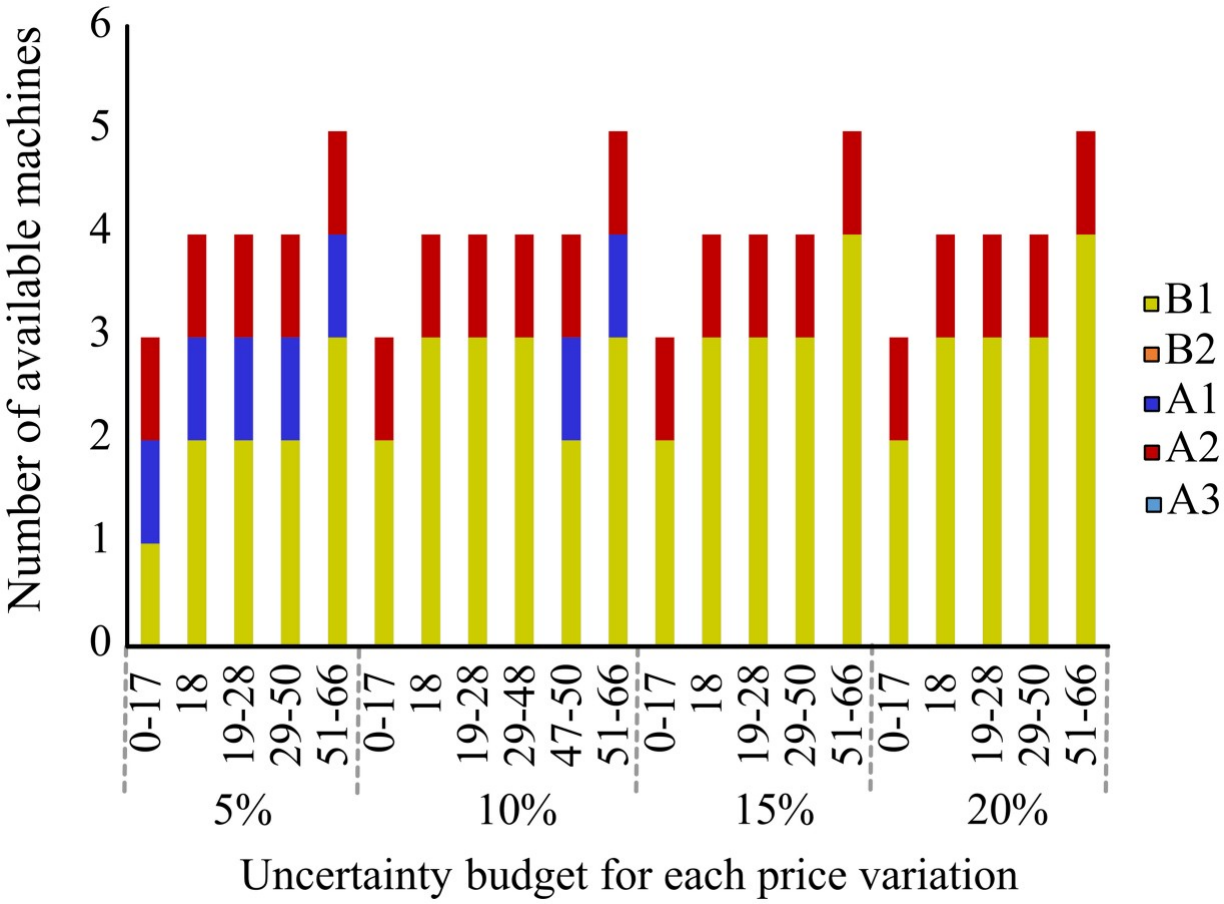
Machinery configuration for variation on A1 price from 0-20%.

To analyze how the cost of each technology affects the acquisition decision, we will take the technology of machine A1, which is preferred in by both models, and study the impact of changing its price between 5-20%, without changing the cost of the other technologies. In Fig 10 we can observe how the acquisition decision is affected in the RO model under different uncertainty budgets and percentage change of technology A1. As it can be expected, the optimal solution of the RO model changes the machinery configuration, by acquiring more B1 technology instead of A1. Under small changes in the technology price (e.g., between 5% to 10%), the model still selects at least 1 machine of technology A1, specially under high robustness budgets. But after a 15% in price increase of technology A1, it becomes too high, so the model decides only for B1 technology. These results are in line to what we can observe in Table 10, in here we can see that as the ratio of laser speed versus cost is higher, the technology is preferred among others, as long as we can process all products. If this ratio is altered due to a change in the cost of the technology, so it will be the acquisition decision.

## 6 Conclusion and managerial insights

We have developed and implemented deterministic and robust optimization investment and production models for the laser cutting process service. The goal of the models is to determine the optimal machinery configuration and a production plan which maximizes the company’s benefits under demand uncertainty. The decision-maker can choose *a priori* the robustness level, which will match their risk aversion. It is essential to highlight that a higher robustness level means more protection in case of uncertainty, but it usually has a higher cost.

We implemented the model using actual production data from a company that provides laser cutting services and performed a sensitivity analysis to find the optimal level of robustness. Results show that the Robust Optimization model renders higher economic benefits than the deterministic linear optimization model under the presence of demand variability. The addition of uncertainty in the model leads to more realistic solutions which can be used to make strategic decisions. The RO model can deliver robust solutions which are less sensitive to the parameters’ variation. The sensitivity analysis shows that the optimal solution is mainly affected by the level of uncertain demand and the cost of the laser cutting equipment. Furthermore, changes in those variables highly impact the company’s economic benefits.

As the demand variability increases, the optimal technology acquisition and production plan of the deterministic (LO) approach does not have enough capacity to process the entire demand. The extra capacity in the RO plan allows the company to be covered and process more orders if demand increases unexpectedly. The higher the uncertainty budget, the higher protection the company has in front of demand variations. This protection is derived from the extra capacity available but is not free of expense. As the robustness budget is increased, the decision-maker may over-invested in its buffer capacity over the demand uncertainty and decreased profits.

As a managerial insight, we can observe that it is interesting to see that the type of technology to acquire depends upon two aspects: the range of products they can process, as a measure of the flexibility of the process, and the cost. As the technology becomes more expensive or the robustness budget is increased, the model selects a different technology portfolio according to the flexibility requirements of the process.

In this work, we have only assumed one source of uncertainty, which is demand. Further research should incorporate other sources of uncertainty, such as cutting process parameters, cost of machinery maintenance, production yield, among others. We have also assumed that the technology costs are fixed during the period. It is common to observe that as technology becomes available mainly, their cost is reduced. Moreover, we have not added the possibility that new and more efficient technology can become available in the future, rendering the current obsolete, changing the time and type of technology to acquire.
